# Specific Interaction of Novel *Friunavirus* Phages Encoding Tailspike Depolymerases with Corresponding Acinetobacter baumannii Capsular Types

**DOI:** 10.1128/JVI.01714-20

**Published:** 2021-02-10

**Authors:** A. V. Popova, M. M. Shneider, N. P. Arbatsky, A. A. Kasimova, S. N. Senchenkova, A. S. Shashkov, A. S. Dmitrenok, A. O. Chizhov, Y. V. Mikhailova, D. A. Shagin, O. S. Sokolova, O. Y. Timoshina, R. S. Kozlov, K. A. Miroshnikov, Y. A. Knirel

**Affiliations:** a Moscow Institute of Physics and Technology (National Research University), Dolgoprudny, Moscow Region, Russia; b State Research Center for Applied Microbiology and Biotechnology, Obolensk, Moscow Region, Russia; c Institute of Antimicrobial Chemotherapy, Smolensk State Medical University, Smolensk, Russia; d Shemyakin-Ovchinnikov Institute of Bioorganic Chemistry, Russian Academy of Sciences, Moscow, Russia; e Zelinsky Institute of Organic Chemistry, Russian Academy of Sciences, Moscow, Russia; f Central Scientific Research Institute of Epidemiology, Moscow, Russia; g Pirogov Russian National Research Medical University, Moscow, Russia; h Lomonosov Moscow State University, Moscow, Russia; University of Texas Southwestern Medical Center

**Keywords:** bacteriophage, *Acinetobacter baumannii*, tailspike, depolymerase, glycosidase, capsular polysaccharide, capsular type, bacteriophage

## Abstract

Acinetobacter baumannii is one of the most clinically important nosocomial pathogens. The World Health Organization refers it to its “critical priority” category to develop new strategies for effective therapy. This microorganism is capable of producing structurally diverse capsular polysaccharides (CPSs), which serve as primary receptors for A. baumannii bacteriophages carrying polysaccharide-degrading enzymes. In this study, eight novel bacterial viruses that specifically infect A. baumannii strains belonging to K2/K93, K32, K37, K44, K48, K87, K89, and K116 capsular types were isolated and characterized. The overall genomic architecture demonstrated that these viruses are representatives of the *Friunavirus* genus of the family *Autographiviridae*. The linear double-stranded DNA phage genomes of 41,105 to 42,402 bp share high nucleotide sequence identity, except for genes encoding structural depolymerases or tailspikes, which determine the host specificity. Deletion mutants lacking N-terminal domains of tailspike proteins were cloned, expressed, and purified. The structurally defined CPSs of the phage bacterial hosts were cleaved with the specific recombinant depolymerases, and the resultant oligosaccharides that corresponded to monomers or/and dimers of the CPS repeats (K units) were isolated. Structures of the derived oligosaccharides were established by nuclear magnetic resonance spectroscopy and high-resolution electrospray ionization mass spectrometry. The data obtained showed that all depolymerases studied were glycosidases that specifically cleave the A. baumannii CPSs by the hydrolytic mechanism, in most cases, by the linkage between the K units.

**IMPORTANCE** Acinetobacter baumannii, a nonfermentative, Gram-negative, aerobic bacterium, is one of the most significant nosocomial pathogens. The pathogenicity of A. baumannii is based on the cooperative action of many factors, one of them being the production of capsular polysaccharides (CPSs) that surround bacterial cells with a thick protective layer. Polymorphism of the chromosomal capsule loci is responsible for the observed high structural diversity of the CPSs. In this study, we describe eight novel lytic phages which have different tailspike depolymerases (TSDs) determining the interaction of the viruses with corresponding A. baumannii capsular types (K types). Moreover, we elucidate the structures of oligosaccharide products obtained by cleavage of the CPSs by the recombinant depolymerases. We believe that as the TSDs determine phage specificity, the diversity of their structures should be taken into consideration as selection criteria for inclusion of certain phage candidates in the cocktail designed to control A. baumannii with different K types.

## Abstract (precis)

Acinetobacter baumannii, a nonfermentative, Gram-negative, aerobic bacterium, is one of the most significant nosocomial pathogens. The pathogenicity of A. baumannii is based on the cooperative action of many factors, one of them being the production of capsular polysaccharides (CPSs) that surround bacterial cells with a thick protective layer.

## INTRODUCTION

Hospital-acquired or nosocomial infections are the most frequent complications in hospitalized patients and the major reason for prolongation of hospital stays and mortality. Acinetobacter baumannii is one of the most significant nosocomial pathogens; it is characterized by intrinsic and acquired resistance to different antibiotics and disinfectants, tolerance to antiseptics and detergents, UV irradiation, and drying, and the ability to form biofilms on various biotic and abiotic surfaces (1, 2). A current prominent problem is the rapidly growing resistance of nosocomial A. baumannii strains to carbapenems, the drugs of choice for the treatment of severe hospital-acquired infections. In particular, resistance rates to carbapenems exceed 80% in nosocomial A. baumannii in Russia, and almost 25% of isolates are resistant to all clinically available antibiotics except colistin (3).

Application of lytic bacteriophages and phage-derived enzymes is one of the possible approaches to control the spread of multidrug-resistant A. baumannii strains. Bacteriophages are natural regulators of the population of microorganisms, their diversity in nature is extremely high, and they use a number of strategies to infect and destroy bacteria (4). The ability of bacteriophages to infect bacterial cells, first of all, depends on their ability to recognize specific determinants on the bacterial surface. Over recent years, a number of research groups have demonstrated that capsular polysaccharides (CPSs) are the primary receptors for A. baumannii bacteriophages carrying polysaccharide-degrading enzymes (5–9). The polymorphism of the chromosomal capsule loci (K loci [KL]) is responsible for the observed high diversity of CPS structures (10–12). To date, more than 140 KL variants have been identified by analysis of A. baumannii genome sequences (J. J. Kenyon, Queensland University of Technology, Brisbane, Australia, personal communication), and the CPS structures have been established for more than 40 capsular types (K types).

To our knowledge, among all publications devoted to A. baumannii polysaccharide-degrading enzymes, there are only two studies that describe the mechanisms of A. baumannii CPS cleavage by phage depolymerases. In particular, it has been established how the tailspike depolymerase (TSD) of podophage φAB6 hydrolyzed the CPS of A. baumannii clinical isolate Ab-54149, and the CPS-digested products were identified by one- and two-dimensional nuclear magnetic resonance (NMR) spectroscopy (6). In our previous work, we studied the interaction of depolymerases of four lytic phages with the corresponding A. baumannii CPSs (13). TSDs of three bacteriophages, Fri1, AS12, and BS46, were demonstrated to be specific glycosidases that cleave the CPSs of A. baumannii strains 28 (K19 capsular type), 1432 (K27), and B05 (K9), respectively, by the hydrolytic mechanism. The TSD of bacteriophage AP22 was characterized as a polysaccharide lyase that cleaved the CPS of A. baumannii strain 1053 (K91) by β-elimination in hexuronic acid (d-ManNAcA) residues.

Herein, we present a characterization of several novel bacteriophages that infect A. baumannii strains belonging to K2/K93, K32, K37, K44, K48, K87, K89, and K116 capsular types and elucidate the mechanisms of specific CPS cleavage by depolymerases encoded in their genomes. We believe that the detailed characterization of new lytic phages and phage-encoded depolymerases will expand our understanding of virus-bacterial host interaction strategies. Most of all, within the scope of possible phage application, the search of lytic phages with different depolymerases seems to be the most reasonable approach to personalized medicine, when an antibacterial agent is selected strictly to the A. baumannii capsular types circulating in a particular location/hospital.

## RESULTS

### Phage isolation, morphology, and host specificity.

Bacteriophages vB_AbaP_APK2, APK32, APK37, APK44, APK48, APK87, APK89, and APK116 were initially isolated on the bacterial lawns of A. baumannii ACICU (K2), LUH5549 (K32), NIPH146 (K37), NIPH70 (K44), NIPH615 (K48), LUH5547 (K87), LUH5552 (K89), and MAR303 (K116) from sewage and environmental (river water) samples by using an enrichment procedure in 2018. The phages were named according to a rational scheme for the nomenclature of viruses of *Bacteria* and *Archaea* (14), where APK is a short laboratory name which means Acinetobacter phage and the number of the K type that is infected by the phage, for example, APK2 indicates the phage isolated on the bacterial lawn of A. baumannii ACICU belonging to K2.

**FIG 1:**
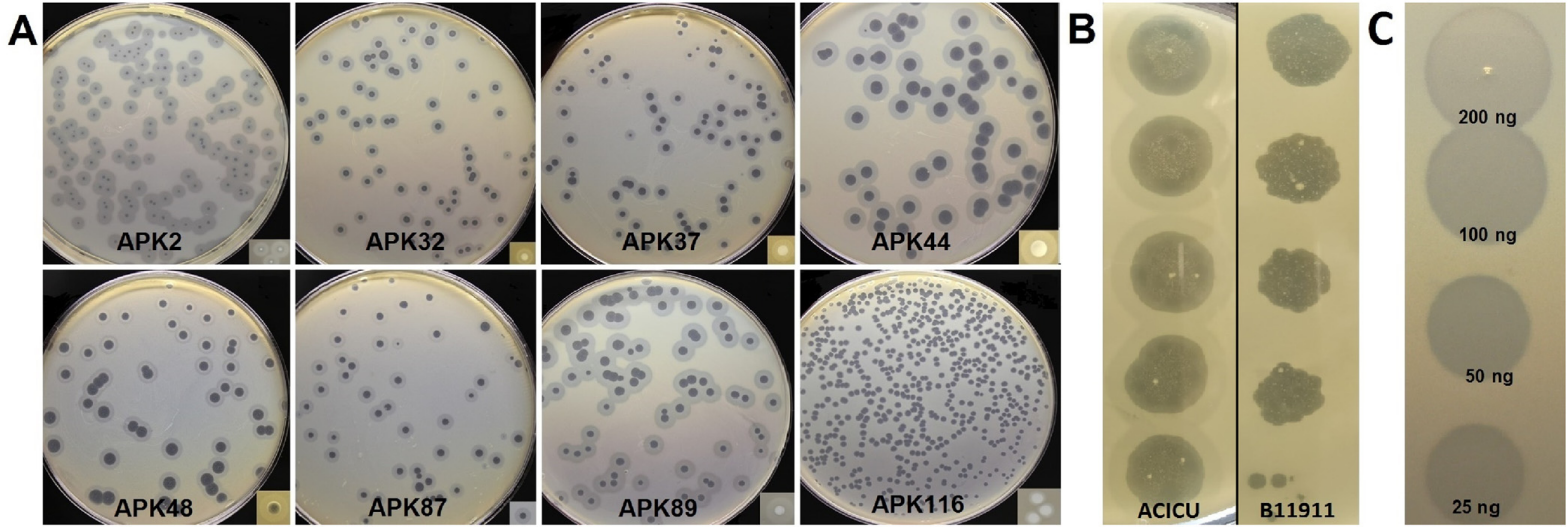
(A) Phage plaques with opaque haloes formed by phages APK2, APK32, APK37, APK44, APK48, APK87, APK89, and APK116 on A. baumannii ACICU (K2), LUH5549 (K32), NIPH146 (K37), NIPH70 (K44), NIPH615 (K48), LUH5547 (K87), LUH5552 (K89), and MAR303 (K116), respectively. (B) Phage APK2 spot titration from the top down (10-fold serial dilutions) on lawns of A. baumannii ACICU and B11911 after overnight incubation. (C) Spot test with serial 2-fold titration of purified recombinant depolymerase APK32_gp46 on A. baumannii LUH5549 lawn after 8 h of incubation.

On the lawns of A. baumannii host strains, the phages formed clear plaques surrounded by haloes (Fig. 1A), indicating the presence of phage structural depolymerases degrading polysaccharide capsules (5–9, 15).

**FIG 2:**
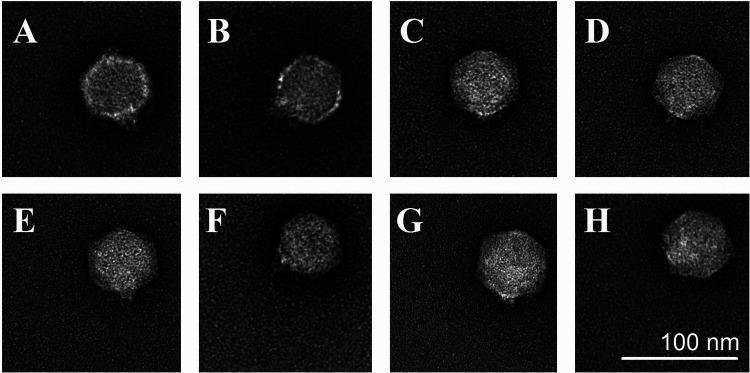
Transmission electron micrographs of phages APK2 (A), APK32 (B), APK37 (C), APK44 (D), APK48 (E), APK87 (F), APK89 (G), and APK116 (H). Staining with 1% uranyl acetate. Scale bar, 100 nm.

As examined by transmission electron microscopy (TEM), all the phages had icosahedral heads of approximately 60 nm in diameter and short noncontractile tails of 10 nm in length (Fig. 2).

The host specificity of phages APK2, APK32, APK37, APK44, APK48, APK87, APK89, and APK116 was tested using a collection of A. baumannii strains belonging to 56 different K types (Table 1). K loci identified in the genomes of these strains were annotated earlier, and CPS structures for most of them were biochemically characterized. It was found that all of the phages except for APK2 were highly specific and able to infect only a strain of a certain K type. Phage APK2 could infect the representatives of two capsular types, namely, A. baumannii ACICU (K2) and A. baumannii B11911 (K93) (Fig. 1B). In return, phage APK93 isolated from another sample of wastewater on the bacterial lawn of A. baumannii B11911 (K93) was able to infect A. baumannii ACICU (K2). Based on this observation, we suggest that structural depolymerases of these phages recognize and degrade a similar linkage in the CPS structures of A. baumannii strains ACICU and B11911.

**TABLE 1:** Acinetobacter baumannii strains used in this study for phage specificity determination

K type	A. baumannii strains with confirmed CPS structure	K locus GenBank accession no. or coordinates within whole-genome shotgun sequences	Reference or source
1	AYE	CU459141 (base position range, 3834156–3863316)	10
2	ACICU	CP000863 (base position range, 88768–115030)	10, 11, 18
3/22^*a*^	ATCC 17978/LUH5537	CP000521 (base position range, 56835–79908)/ KC526920	10, 20/11, 20
6	RBH4	KF130871	46
7	LUH5533	KC526894	11, 47
8	BAL097	KX712116	48
9	B05	MK331712	9
11	LUH5545	KC526904	11, 49
15	LUH5554	KC526900	11, 50
16	D4	MF522813	51
17	G7	KC118541	52
19	28	KU215659	53
20	A388	JQ684178	54
21	G21	MG231275	54
24	RCH51	KX756650	44
25	AB5075	BK008886	55
27	4190	KT266827	21
30	NIPH190	MN166189	22
32	LUH5549	KC526897	11, 19
33	NIPH67	MN166195	56
35	LUH5535	KC526896	50
37	NIPH146	APOU01000009 (base position range, 32574–53092)	20
42	LUH5550	KC526903	57
43	LUH5544	KC526905	11, 58
44	NIPH70	APRC01000043 (base position range, 97989–129118)	21
45	NIPH201	MN166190	22
46	NIPH329	MK609549	59
47	NIPH601	MN166193	58
48	NIPH615	MN166191	22
51	WM98b	MN148384	Unpublished
52	LUH5546	KC526899	11
53	D23	MH190222	60
54	RCH52	MG867726	48
55	BAL204	MN148381	61
57	BAL212	KY434631	62
58	BAL114	KT359617	Unpublished
61	NL4	To be registered	J. Kenyon, personal communication
73	SGH0703	MF362178	63, 64
74	BAL309	MN148383	61
80	LUH3712	KC526914	11, 44
81	LUH3713	KC526916	11
82	LUH5534	KC526908	11, 65
83	LUH5538	KC526898	11, 49
84	LUH5540	KC526902	11
85	LUH5543	KC526913	61
87	LUH5547	KC526918	11; this research
88	LUH5548	KC526910	11, 58
89	LUH5552	KC526919	11; this research
90	LUH5553	KC526917	11
91	1053	KM402814	66
92	B8300	CP021347 (base position range, 1420707–1451977)	Unpublished
93	B11911	BK010902	23
116	MAR-303	MK399425	24
125	MAR13-1452	MH306195	67
128	KZ-1093	MK399428	68

a These KL clusters are closely related, although they are slightly different at the nucleotide level, but produce CPSs with identical structures.

### Phage genome organization and comparison.

The phage linear genomes ranged from 41,105 to 42,402 bp in size, with direct terminal repeats (DTRs) of 381 to 417 bp at the genomes’ ends, containing between 49 and 54 predicted genes located only on the forward strands (Table 2). The G+C content of the genomes was 39.05% to 39.40%, similar to that of other A. baumannii viruses and close to the approximate average values for different A. baumannii strains (38.94% to 39.4% according to reference 16). No tRNA genes were identified. No genes encoding toxins and no products responsible for antibiotic resistance or related to lysogeny were determined in the phage genomes.

**TABLE 2:** General characteristics of phage genomes and phage-encoded TSDs

Phage name	Genome length (bp)	G+C content (%)	Total no. of genes	DTR length (bp)	GenBank accession no.	Phage-encoded depolymerases	
						Gene (protein ID)	Protein size (aa)
APK2	41,476	39.24	50	410	MK257719	APK2_43 (AZU99242.1)	693
APK32	41,142	39.31	52	396	MK257722	APK32_46 (AZU99395.1)	678
APK37	41,981	39.16	51	388	MK257723	APK37_44 (AZU99445.1)	822
APK44	41,461	39.07	50	382	MN604238	APK44_44 (QGK90444.1)	752
APK48	41,105	39.26	49	417	MN294712	APK48_43 (QFG06960.1)	740
APK87	42,402	39.05	54	381	MN604239	APK87_48 (QGK90498.1)	720
APK89	41,198	39.40	52	397	MN651570	APK89_46 (QGK90394.1)	748
APK116	41,765	39.08	49	409	MN807295	APK116_43 (QHS01530.1)	861

Almost all of the predicted proteins encoded by APK2, APK32, APK37, APK44, APK48, APK87, APK89, and APK116 had very close homologues in the genomes of phages belonging to the genus *Friunavirus* of the subfamily *Beijerinckvirinae* (recently designated by the International Committee on Taxonomy of Viruses [ICTV]), within the family *Autographiviridae*.

Based on the functions of homologous proteins, early, middle, and late gene regions were identified in APK2, APK32, APK37, APK44, APK48, APK87, APK89, and APK116 genomes, as well as in the genome of phage Fri1 and other Fri1-like viruses, such as vB_AbaP_AS11 and vB_AbaP_AS12 (7).

The functions of products encoded by genes of early regions (left parts of the genomes) were not determined, but it is likely that these proteins are involved in inhibiting or redirecting the activities of functionally important host system components to serve the needs of the viruses (17).

The middle regions of the phage genomes comprised nucleotide metabolism and DNA replication and repair genes encoding DNA primase, DNA helicase, ATP-dependent ligase, DNA polymerase I, 5′–3′ exonuclease, tRNA nucleotidyltransferase, endonuclease VII, phosphoesterase, deoxynucleotide monophosphate kinase, and also the gene encoding single-subunit viral RNA polymerase. Interestingly, genes encoding tRNA nucleotidyltransferases were absent in the genomes оf phages APK32, APK37, and APK48 and thus, most likely, are not essential for phage nucleotide metabolism. The presence of open reading frames (ORFs) encoding putative HNH endonucleases was also revealed in the middle clusters of all studied phages, exсept for phages APK89 and APK116. Some homing endonuclease genes were found immediately upstream (APK87_*g*25) or downstream (APK2_*g*22, APK37_*g*23, APK44_*g*23, APK48_*g*21) of the DNA polymerase I gene. In some cases, HNH endonuclease genes (APK32_*g*24, APK44_*g*21) interrupted the DNA polymerase gene into the two domains, and in some cases, they were located between genes encoding 5′–3′ exonuclease and DNA endonuclease VII (APK32_g29, APK48_g26) instead of genes encoding tRNA nucleotidyltransferase.

**FIG 3:**
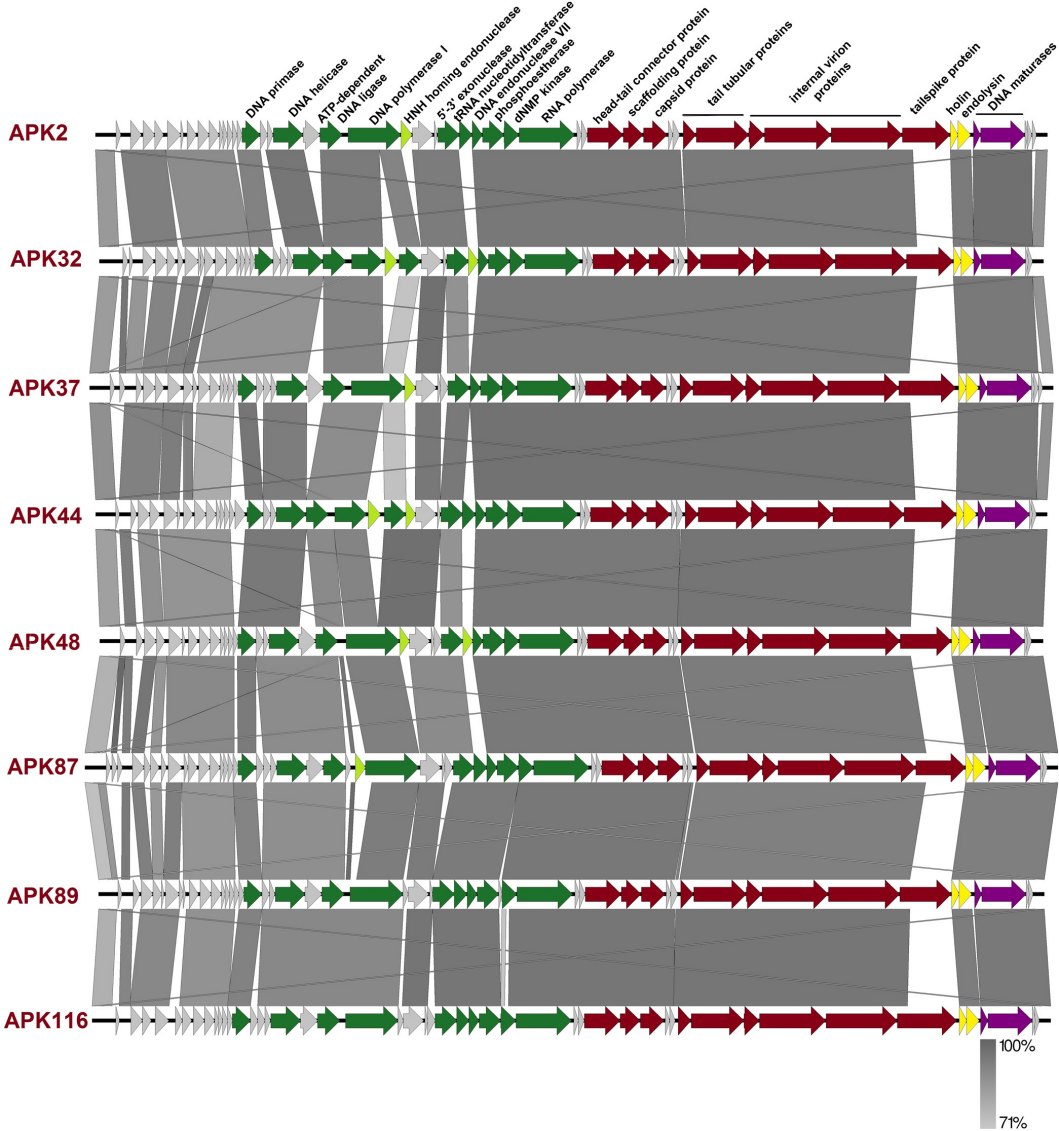
Comparison of phage genomes using the following color scheme: light gray, genes with unknown functions; green, genes encoding enzymes of nucleotide metabolism, proteins involved in DNA replication and repair, and RNA polymerase; red, structural protein genes; yellow, genes encoding proteins associated with lysis; violet, DNA-packaging protein genes. Maps were created with Easyfig.

Late genome regions were highly conserved among all Fri1-like viruses, containing genes encoding structural proteins (head-tail connector protein, scaffolding protein, major capsid protein, tail tubular proteins A and B, internal virion proteins A, B, and C, and tailspike), proteins associated with bacterial cell lysis (holin and endolysin), and with proteins associated with the packaging of DNA (DNA maturase A and B) (Fig. 3).

The most variable regions of the phage genomes were among the early genes, genes encoding some hypothetical proteins, and gene regions encoding CPS-recognizing/degrading domains of phage tailspikes or structural depolymerases.

BLAST analysis revealed that the closest nucleotide sequence homologs of APK2 were phages APK93 (GenBank accession no. MK257721), APK-2 (MK257720), IME200 (NC_028987), and vB_AbaP_AGC01 (MT263719). For these, the genome coverages obtained to an E-value of 0.0 were 99%, 99%, 96%, and 96%, with identities of 99.23%, 99.04%, 96.71%, and 94.69%, respectively. The phages APK32, APK37, APK44, APK48, APK87, APK89, and APK116 shared the highest percentage of DNA similarity with phages vB_AbaP_B1 (NC_042003), APK44, APK37, APK116, vB_ApiP_P2 (NC_042007), vB_AbaP_AS11 (NC_041915), and APK48, respectively.

### Phage depolymerases.

Recently, it has been shown that tailspike proteins or structural depolymerases of A. baumannii phages direct the ability to infect the strains of certain K types (5–9). Depolymerases are highly specific enzymes that cleave CPS of definite structure for subsequent phage adsorption on the bacterial cell surface (15).

Tailspikes of phages APK2, APK32, APK37, APK44, APK48, APK87, APK89, and APK116 were formed by single proteins encoded by the genes located at the end of structural modules of phage genomes immediately after internal virion proteins A to C (Fig. 3; Table 2).

At the amino acid level, APK32, APK37, APK44, APK48, APK87, APK89, and APK116 TSDs were found to differ significantly from the proteins encoded by the other A. baumannii phages deposited in GenBank, except for their N-terminal domains (approximately the first 160 amino acids of the proteins). These domains were responsible for the attachment of variable CPS-recognizing/degrading parts of the tailspikes to the tail tubular structures of phage particles and, as expected, were very conservative within the phages of the same taxonomic group sharing similar morphology and structural components.

BLASTp analysis revealed that APK2_gp43 was identical to the proteins encoded by phages vB_AbaP_APK2-2 (APK2-2_gp43; accession no. AZU99292) and vB_AbaP_APK93 (APK2_gp43; GenBank accession no. AZU99342) and was highly homologous to the proteins encoded by Acinetobacter phage IME200 (YP_009216489; 99% at the amino acid level) and Acinetobacter phage SH-Ab 15519 (YP_009598268; 97% at the amino acid level). This, most likely, indicates that tailspikes of phages APK2, IME200, and SH-Ab 15519 can interact specifically with CPS of the same structure.

Deletion mutants lacking the N-terminal domains of the tailspikes were cloned, expressed, and purified by immobilized metal ion affinity chromatography, followed by ion-exchange chromatography. The recombinant proteins were stable for at least 2 months at 4°C, retaining sufficient depolymerase activities. An example of serial 2-fold titration of one of the purified recombinant depolymerases on the bacterial lawn of a host strain, after 8 h of incubation, is presented in Fig. 1C.

The spectra of depolymerase activity of recombinant proteins were tested against a panel of A. baumannii strains with the confirmed CPS structures (Table 1) belonging to 56 different K types. All purified recombinant depolymerases, except for APK2_gp43, were found to be highly specific and formed opaque haloes only on the bacterial lawns of A. baumannii strains of the corresponding K types. APK2_gp43 formed haloes on the bacterial lawns of K2 and K93 strains (data not shown), meaning that this depolymerase effectively degrades CPSs of both types.

**FIG 4:**
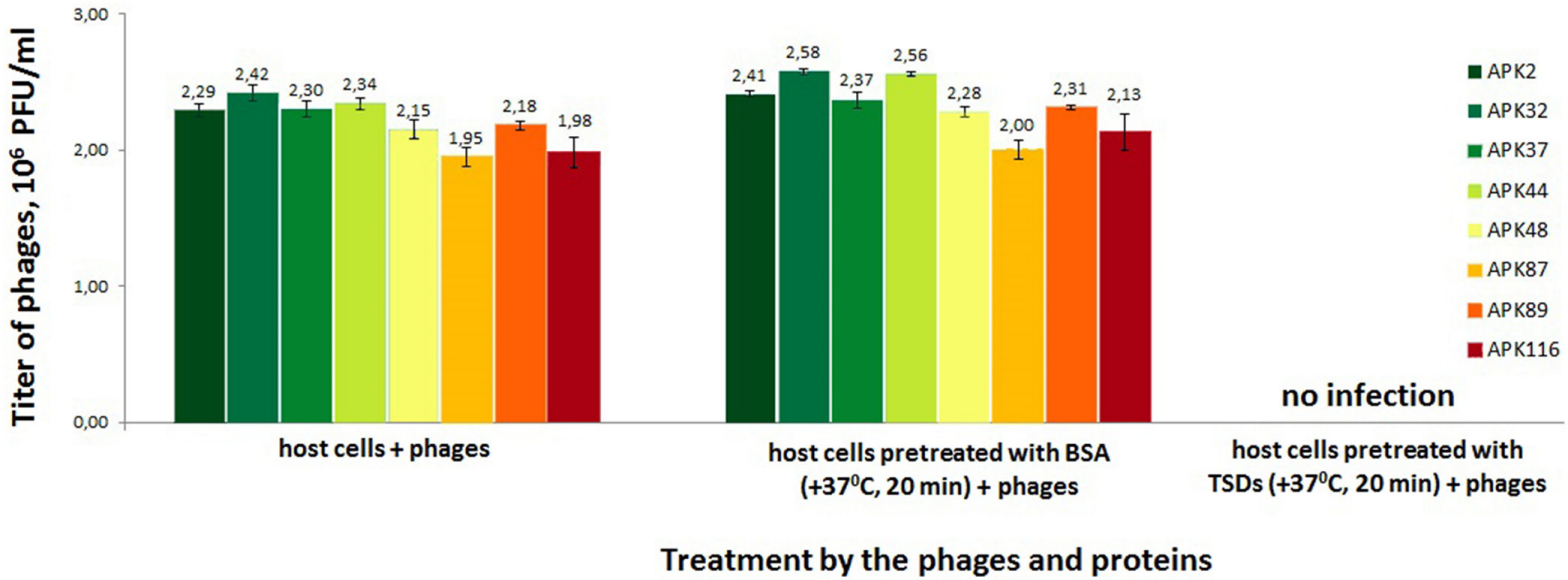
Phage infection inhibition by the TSDs. Phage titers observed on the bacterial lawns after the treatment of A. baumannii host cells with phages only (left series), host cell cultures preincubated with BSA (as a negative control; middle series), and purified TSDs proteins (right series) followed by phage treatment. Error bars represent 1 standard deviation from the arithmetic mean.

In order to investigate the role of TSDs in phage infection of corresponding A. baumannii host cells, we have conducted a series of competition experiments (Fig. 4). For this, bacterial cell cultures preincubated with purified TSD proteins were mixed with several phage dilutions and plated on agar dishes. After overnight incubation, phage titers were measured. A negative-control experiment series where phage host bacterial cells were pretreated with bovine serum albumin (BSA) showed no significant differences in phage titers, whereas coincubation with APK2_gp43, APK32_gp46, APK37_gp44, APK44_gp44, APK48_gp43, APK87_gp48, APK89_gp46, and APK116_gp43 resulted in A. baumannii host cells becoming nonsusceptible to infection by the corresponding phages. Thus, the addition of 20 μM TSD proteins to the cells completely inhibits plaque formation. This means that the TSDs effectively degraded capsular polysaccharide layers surrounding A. baumannii host cells and, after that, specific phages could not adsorb to the cells. The results obtained confirm that the CPSs are the primary receptors for the phages and that TSDs play a crucial role in the initial step of the phage-bacterial cell interaction.

### Structures of the CPSs of A. baumannii phage host strains.

To elucidate mechanisms of the action of phage depolymerases, structures of the phage host CPSs, which are the primary receptors for depolymerase-carrying bacteriophages, were characterized.

**FIG 5:**
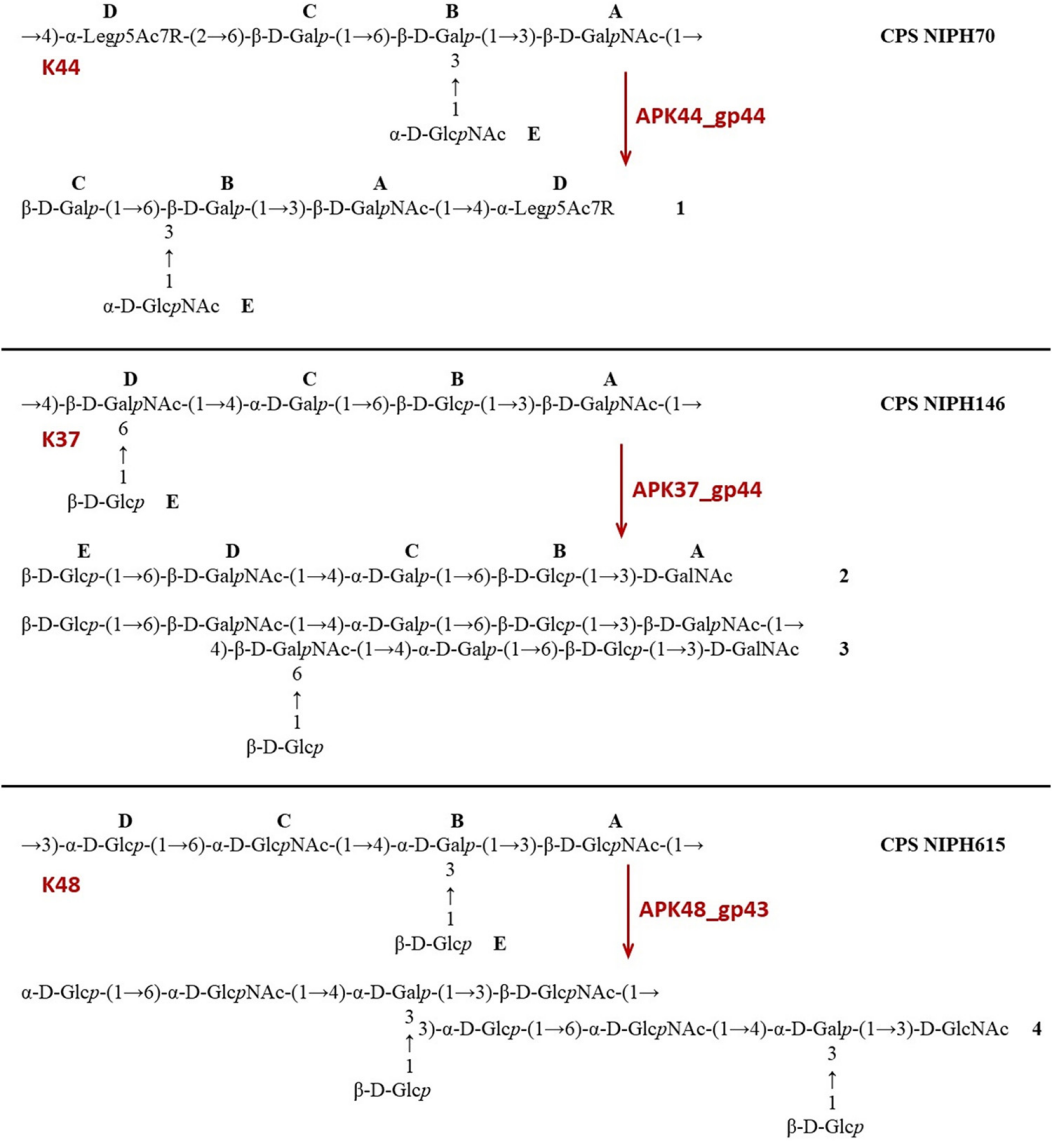
Structures of the CPSs of A. baumannii NIPH70 (K44), NIPH146 (K37), NIPH615 (K48), and oligosaccharides **1** to **4** derived by depolymerization of the CPSs with phage TSDs (see also Table 3). In the NIPH70 (K44) CPS and oligosaccharide **1**, R indicates *N*-acetyl or *N*-[(*S*)-3-hydroxybutanoyl] (∼1:2.5).

**FIG 6:**
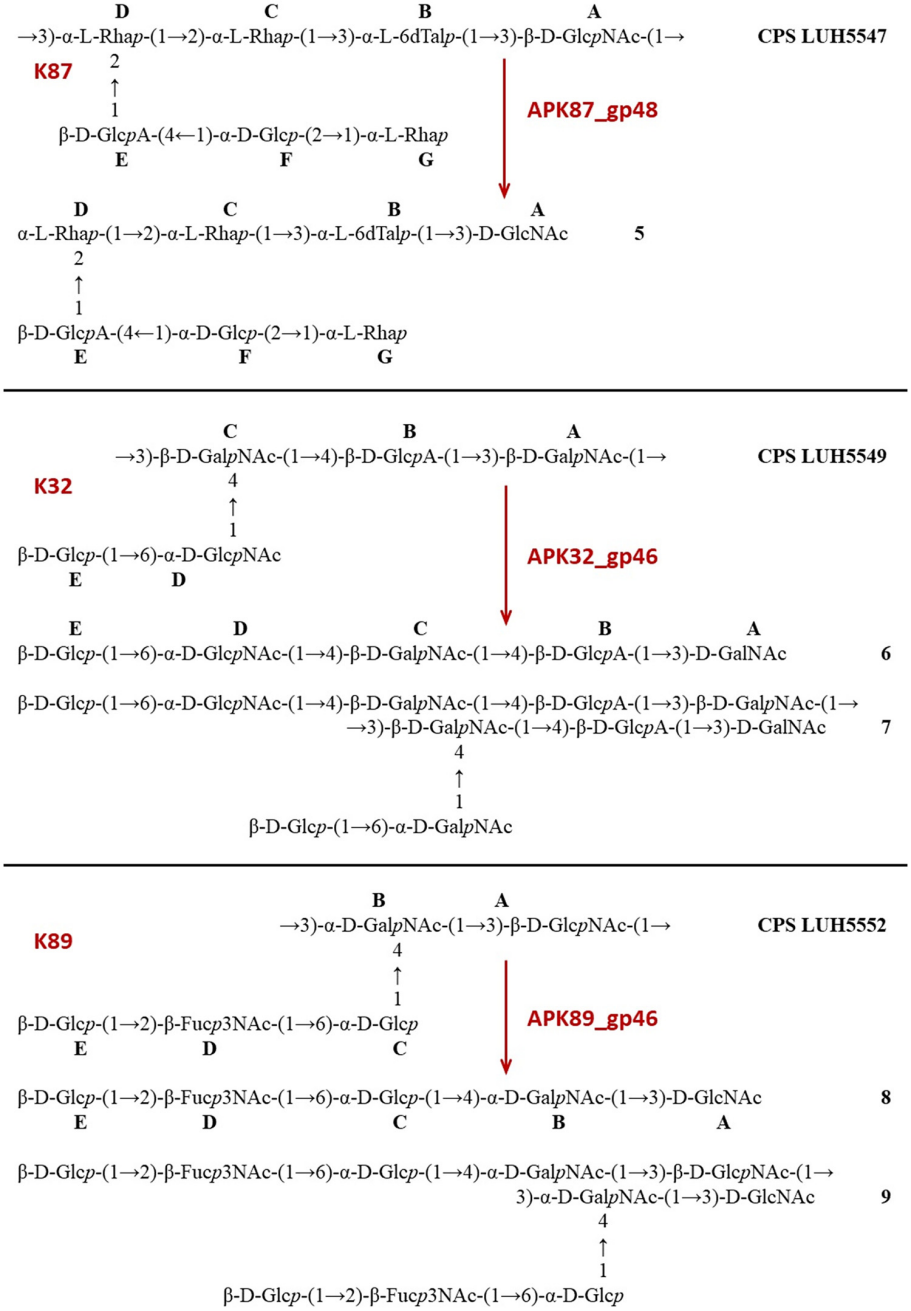
Structures of the CPSs of A. baumannii LUH5547 (K87), LUH5549 (K32), and LUH5552 (K89) and oligosaccharides **5** to **9** derived by depolymerization of the CPSs with phage TSDs (see also Table 3). (∼10% K units of the CPS of LUH5547 and oligosaccharide 5 carry an *O*-acetyl group at an unknown position).

**FIG 7:**
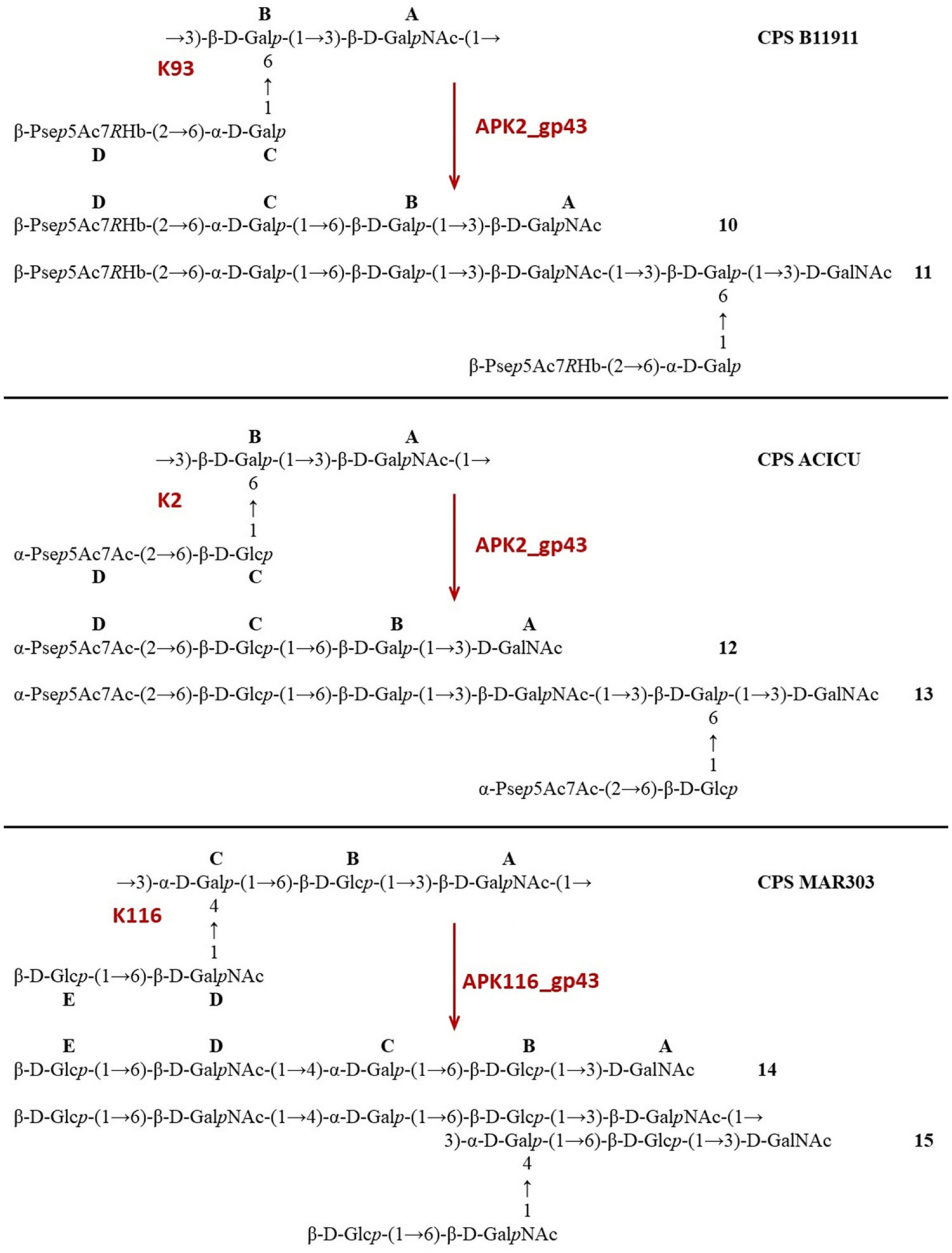
Structures of the CPSs of A. baumannii B11911 (K93), ACICU (K2), MAR303 (K116), and oligosaccharides **10** to **15** derived by depolymerization of the CPSs with phage TSDs (for details, see Table 3).

CPSs were isolated by phenol-water extraction from A. baumannii strains ACICU (18), LUH5549 (19), NIPH146 (20), NIPH70 (21), NIPH615 (22), LUH5547, LUH55552, B11911 (23), and MAR303 (24), belonging to K2, K32, K37, K44, K48, K87, K89, K93, and K116 capsular types, respectively. The structures of the K2, K32, K37, K44, K48, K93, and K116 CPSs were established earlier (Fig. 5–7). They are built up of tetrasaccharide (K2 and K93) or pentasaccharide (the other CPSs) repeats (K units) containing mainly d-Glc, d-Gal, d-GlcNAc, and d-GalNAc (common monosaccharides). The K2, K44, and K93 CPSs of strains ACICU, NIPH70, and B11911 also include derivatives of higher aldulosonic acids: 5,7-diamino-3,5,7,9-tetradeoxy-l-glycero-l-manno-non-2-ulosonic (pseudaminic) acid (Pse) or 5,7-diamino-3,5,7,9-tetradeoxy-d-glycero-d-galacto-non-2-ulosonic (legionaminic) acid (Leg).

Structures of the K87 and K89 CPSs of A. baumannii LUH5547 and LUH5552, respectively, have not been reported earlier but, according to our unpublished data, are as shown in Fig. 6. The CPS of strain LUH5552 has a pentasaccharide K unit, which in addition to common monosaccharides contains 3-acetamido-3,6-dideoxy-d-galactose (Fuc3NAc). The CPS of LUH5547 is distinguished by the presence of d-GlcA, 6-deoxy-l-talose (l-6dTal), and three residues of l-rhamnose (l-Rha) in a heptasaccharide K unit. Details of the structure elucidation of these CPSs will be reported elsewhere.

### Mechanism of cleavage of A. baumannii CPSs by phage depolymerases.

The purified CPSs of ACICU/B11911, LUH5549, NIPH146, NIPH70, NIPH615, and MAR303 were cleaved with recombinant depolymerases APK2_gp43, APK32_gp46, APK37_gp44, APK44_gp44, APK48_gp43, APK87_gp48, APK89_gp46, and APK116_gp43, and oligosaccharide products were fractionated by Fractogel TSK HW-40S gel permeation chromatography (Table 3). The CPSs of strains NIPH70 and LUH5547 gave a single oligosaccharide each (**1** and **5**, respectively, which corresponded to the K units [Fig. 5 and 6]). No K unit monomer, but a dimer **4**, was obtained from the CPS of strain NIPH615 (Fig. 5). Each of the other CPSs afforded both a monomer and a dimer of the K unit: **2** and **3** from NIPH146 (Fig. 5), **6** and **7** from LUH5549, **8** and **9** from LUH5552 (Fig. 6), **10** and **11** from B11911, **12** and **13** from ACICU, and **14** and **15** from MAR303 (Fig. 7). All CPSs of the last group also gave K unit trimers and higher products.

**TABLE 3:** Cleavage of A. baumannii CPSs with specific TSDs

Phage	Depolymerase	A. baumannii strain	CPS type	Linkage in the CPS that is cleaved by a specific depolymerase^*a*^	Data for depolymerization products			
					Monomer	Dimer	Structures	NMR data
APK44	gp44	NIPH70	K44	α-Leg*p*5Ac7R-(2→6)-β-d-Gal*p* (**D→C**)	**1**		Fig. 5	Table 5
APK37	gp44	NIPH146	K37	β-d-Gal*p*NAc-(1→4)-β-d-Gal*p*NAc (**A→D**)	**2**	**3**	Fig. 5	Table 6
APK48	gp43	NIPH615	K48	β-d-Glc*p*NAc-(1→3)-α-d-Glc*p* (**A→D**)		**4**	Fig. 5	
APK87	gp48	LUH5547	K87	β-d-Glс*p*NAc-(1→3)-α-l-Rha*p* (**A→D**)	**5**		Fig. 6	Table 7
APK32	gp46	LUH5549	K32	α-d-Gal*p*NAc-(1→3)-β-d-Gal*p*NAc (**A→C**)	**6**	**7**	Fig. 6	Table 8
APK89	gp46	LUH5552	K89	β-d-Glc*p*NAc-(1→3)-α-d-GalNAc*p* (**A→B**)	**8**	**9**	Fig. 6	Table 9
APK2	gp43	B11911	K93	β-d-Gal*p*NAc-(1→3)-β-d-Gal*p* (**A→B**)	**10**	**11**	Fig. 7	Table 10
APK2	gp43	ACICU	K2	β-d-Gal*p*NAc-(1→3)-β-d-Gal*p* (**A→B**)	**12**	**13**	Fig. 7	
APK116	gp43	MAR303	K116	β-d-Gal*p*NAc-(1→3)-α-d-Gal*p* (**A→C**)	**14**	**15**	Fig. 7	Table 11

a For full CPS structures, see Fig. 5 to 7.

Structures of the oligosaccharides obtained were established by one- and two-dimensional ^1^H and ^13^C NMR spectroscopy (25) and were confirmed by high-resolution electrospray ionization mass spectrometry (HR ESI-MS) (Table 4). All oligosaccharides had the same monosaccharide composition as the CPSs they were derived from.

**TABLE 4:** HR ESI-MS data of oligosaccharides **1** to **15** derived by depolymerization of A. baumannii CPSs with specific TSDs^*a*^

Strain	Oligosaccharide	Composition	Molecular mass (Da)	Ion peak at *m/z* (exptl/*calculated*)				
				[M − H]^−^	[M − 2H]^2−^	[M + H]^+^	[M + Na]^+^	[M + K]^+^
NIPH70	1_Ac_	Hex_2_HexN_2_Non_1_Ac_4_	1,064.4020	1,063.3946/*1,063.3947*				
	1_Hb_	Hex_2_HexN_2_Non_1_Ac_3_Hb_1_	1,108.4282	1,107.4206/*1,107.4209*				
NIPH146	2	Hex_3_HexN_2_Ac_2_	910.3278			911.3379/*911.3351*	933.3152/*933.3170*	949.2921/*949.2909*
	3	Hex_6_HexN_4_Ac_4_	1,802.6450				1,825.6307/*1,825.6342*	1,841.5992/*1,841.6082*
NIPH615	**4**	Hex_6_HexN_4_Ac_4_	1,802.6450				1,825.6230/*1,825.6342*	
LUH5547	**5**	Hex_1_6dHex_4_HexA_1_HexN_1_Ac_1_	1,143.4065	1,142.3988/1,142.3992				
LUH5549	**6**	Hex_1_HexA_1_HexN_3_Ac_3_	965.3336			966.3441*/966.3409*	988.3213/*988.3228*	1,004.2890/*1,004.2968*
	**7**	Hex_2_HexA_2_HexN_6_Ac_6_	1912.6566				1,935.6478/*1,935.6458*	
LUH5552	**8**	Hex_2_HexN_2_6dHexN_1_Ac_3_	935.3594				958.3487/*958.3486*	
	**9**	Hex_4_HexN_4_6dHexN_2_Ac_6_	1,852.7083				1,875.6927/*1,875.6975*	
B11911	**10**	Hex_2_HexN_1_Non_1_Hb_1_Ac_2_	905.3488	904.3411/*904.3416*				
	**11**	Hex_4_HexN_2_Non_2_Hb_2_Ac_4_	1,792.6870	1,791.6796/*1,791.6799*	895.3366/*895.3363*			
ACICU	**12**	Hex_2_HexN_1_Non_1_Ac_3_	861.3226	860.3156/*860.3154*			884.3066/*884.3119*	
	**13**	Hex_4_HexN_2_Non_2_Ac_6_	1,704.6346	1,703.6288/*1,703.6274*	851.3184/*851.3101*		1,727.6215/*1,727.6239*	
MAR303	**14**	Hex_3_HexN_2_Ac_2_	910.3326			911.3369/*911.3369*	933.3164/*933.3170*	
	**15**	Hex_6_HexN_4_Ac_4_	1,802.64			1,803.6628/*1,803.6596*		

a Abbreviations: 6dHex, 6-deoxyhexose; Ac, acetyl; Hb, 3-hydroxybutanoyl; Hex, hexose; HexA, hexuronic acid; HexN, 2-amino-2-deoxyhexose; Non, 5,7-diamino-3,5,7,9-tetradeoxynon-2-ulosonic acid.

The ^1^H and ^13^C NMR spectra of the K unit monomers were fully assigned by two-dimensional shift-correlated experiments (^1^H-^1^H correlation spectroscopy [COSY], ^1^H-^1^H total correlation spectroscopy [TOCSY], and ^1^H-^13^C heteronuclear single quantum coherence [HSQC] spectroscopy) and compared with the data of the corresponding CPSs (Tables 5 to 11). Linkage and sequence analyses by two-dimensional ^1^H-^1^H rotating-frame nuclear Overhauser effect (ROESY) and ^1^H-^13^C heteronuclear multiple-bond correlation (HMBC) experiments enabled elucidation of full structures of the oligosaccharides, shown in Fig. 5 to 7.

**TABLE 5:** ^1^H and ^13^C NMR chemical shifts of the K44 CPS of A. baumannii NIPH70 and oligosaccharide **1** derived by depolymerization of the CPS with phage depolymerase APK44_gp44^*a*^

Residue	^1^H and ^13^C chemical shifts (**δ**, ppm)								
	*H-1* C-1	*H-2* C-2	*H-3 (ax, eq)* C-3	*H-4* C-4	*H-5* C-5	*H-6 (a, b)* C-6	*H-7* C-7	*H-8* C-8	*H-9*C-9
CPS (at 30°C) (21)					
→3)-β-d-Gal*p*NAc-(1→ **A**	*4.54* 103.3	*3.93* 52.5	*3.81* 81.8	*4.20* 69.1	*3.67* 75.8	*3.80, 3.80* 62.2			
→3,6)-β-d-Gal*p*-(1→ **B**	*4.45* 106.0	*3.60* 70.3	*3.68* 78.1	*4.09* 66.3	*3.76* 74.5	*3.83, 4.04* 71.0			
→6)-β-d-Gal*p*-(1→ **С**	*4.40* 104.9	*3.48* 71.9	*3.62* 73.9	*3.92* 69.7	*3.76* 74.7	*3.61, 3.89* 64.6			
→4)-α-Leg*p*5Ac7Hb-(2→ **D_Hb_**	174.3	101.7	*1.74, 2.95* 41.4	*3.63* 78.7	*3.75* 51.5	*3.95* 73.1	*3.87* 55.0	*3.94* 68.5	*1.15* 19.5
→4)-α-Leg*p*5Ac7Ac-(2→ **D_Ac_**	174.3	101.7	*1.74, 2.95* 41.4	*3.63* 78.7	*3.75* 51.5	*3.95* 73.1	*3.83* 55.1	*3.94* 68.4	*1.15* 19.4
α-d-Glc*p*NAc-(1→ **E**	*5.02* 95.3	*3.95* 54.7	*3.52* 71.1	*3.81* 72.1	*3.95* 73.1	*3.75, 3.80* 61.6			
Oligosaccharide **1** (at 30°C)						
→4)-β-Leg*p*5Ac7Hb **D_Hb_**			*1.88, 2.42* 40.9	*4.00* 78.0	*3.80* 52.1	*4.23* 70.9	*3.87* 54.3	*3.84* 67.7	*1.15* 20.5
→4)-β-Leg*p*5Ac7Ac **D_Ac_**			*1.88, 2.42* 40.9	*4.00* 78.0	*3.80* 52.1	*4.23* 70.9	*3.83* 54.4	*3.84* 67.7	*1.13* 20.4
→3)-β-d-Gal*p*NAc-(1→ **A**	*4.57* 103.2	*3.91* 52.6	*3.84* 81.8	*4.19* 69.2	*3.67* 75.9	*3.81, 3.81* 62.3			
→3,6)-β-d-Gal*p*-(1→ **B**	*4.47* 105.9	*3.60* 70.3	*3.69* 78.0	*4.11* 66.0	*3.78* 74.5	*3.86, 4.01* 70.3			
β-d-Gal*p*-(1→ **С**	*4.41* 104.7	*3.50* 72.0	*3.63* 74.0	*3.91* 69.9	*3.68* 76.4	*3.75, 3.75* 62.2			
α-d-Glc*p*NAc-(1→ **E**	*5.03* 95.1	*3.96* 54.7	*3.53* 71.0	*3.82* 72.1	*3.97* 73.1	*3.73, 3.80* 61.5			

a Hb, (*S*)-3-hydroxybutanoyl. ^1^H NMR chemical shifts are italicized. Chemical shifts for NHb are δ_C_ 46.1 (C-2), 66.3–66.4 (C-3), and 23.5–23.7 (C-4), δ_H_ 2.36–2.40 (H-2), 4.16–4.18 (H-3), and 1.23–1.24 (H-4); for the *N*-acetyl groups, δ_C_ 23.3–23.7 (CH_3_), δ_H_ 1.95–2.04; for CO of NHb and NAc, 174.3–175.7.

**TABLE 6:** ^1^H and ^13^C NMR chemical shifts of the K37 CPS of A. baumannii NIPH146 and oligosaccharide **2** derived by depolymerization of the CPS with phage depolymerase APK37_gp44^*a*^

Residue	^1^H and ^13^C chemical shifts (**δ**, ppm)					
	*H-1* C-1	*H-2* C-2	*H-3* C-3	*H-4* C-4	*H-5* C-5	*H-6a, 6b* C-6
CPS (at 30°C) (20)						
→3)-β-d-Gal*p*NAc-(1→ **A**	*4.67* 104.4	*4.12* 52.8	*3.85* 81.7	*4.15* 69.2	*3.70* 75.8	*3.81, 3.81* 62.5
→6)-β-d-Glc*p*-(1→ **B**	*4.53* 105.5	*3.31* 74.3	*3.47* 77.0	*3.60* 70.3	*3.60* 75.5	*3.72, 4.01* 66.7
→4)-α-d-Gal*p*-(1→ **C**	*4.96* 99.6	*3.74* 68.8	*3.94* 69.1	*4.36* 77.4	*3.94* 71.4	*3.81, 3.81* 62.1
→4,6)-β-d-Gal*p*NAc-(1→ **D**	*4.91* 102.9	*3.91* 53.6	*3.77* 72.2	*3.91* 80.9	*3.83* 74.7	*3.90, 4.06* 70.2
β-d-Glc*p*-(1→ **E**	*4.48* 104.0	*3.29* 74.3	*3.48* 77.0	*3.39* 71.0	*3.45* 77.1	*3.72, 3.92* 62.1
Oligosaccharide **2** (at 60°C)						
→3)-α-d-GalpNAc **Aα**	*5.24* 92.3	*4.33* 50.0	*4.04* 78.5	*4.20* 69.8	*4.12* 71.3	*3.74-3.78* 62.3
→3)-β-d-GalpNAc **Aβ**	*4.71* 96.3	*3.92* 53.7	*3.89* 81.5	*4.13* 69.1	*3.70* 75.9	*3.74-3.78* 62.1
→6)-β-d-Glcp-(1→ **B**	*4.58*^*b*^ 105.1^*c*^	*3.31* 74.0	*3.49* 76.9	*3.49* 70.7	*4.62* 75.4	*3.78, 3.93* 67.3
→4)-α-d-Galp-(1→ **C**	*4.98* 99.4	*3.73* 69.8	*3.96* 69.0	*4.17* 78.1	*3.93* 71.5	*3.84, 3.84* 62.0
→6)-β-d-GalpNAc-(1→ **D**	*4.67* 103.7	*3.92* 53.8	*3.75* 72.1	*3.97* 70.7	*3.85* 74.6	*3.96, 4.06* 70.5
β-d-Glcp-(1→ **E**	*4.51* 104.0	*3.31* 74.2	*3.51* 76.9	*3.41* 70.9	*3.46* 77.0	*3.75, 3.93* 61.9

a ^1^H NMR chemical shifts are italicized. Chemical shifts for the *N*-acetyl groups are δ_H_ 2.03–2.07, δ_C_ 23.2–23.7 (Me), and 175.8–176.0 (CO).

b When linked to **Aα**; δ 4.52 when linked to **Aβ**.

c When linked to **Aα**; δ 105.3 when linked to **Aβ**.

**TABLE 7:** ^1^H and ^13^C NMR chemical shifts of the *O*-deacetylated LUH5547 (K87) CPS (DPS) and oligosaccharide **5** derived by depolymerization of the CPS with phage depolymerase APK87_gp48^*a*^

Residue	^1^H and ^13^C chemical shifts (**δ**, ppm)					
	*H*-*1* C-1	*H-2* C-2	*H-3* C-3	*H-4* C-4	*H-5* C-5	*H*-*6a,6b* C-6
DPS (at 50°C)^*b*^						
→3)-β-d-Glc*p*NAc-(1→ **A**	*4.75*	*3.85*	*3.66*	*3.53*	*3.49*	*3.78, 3.95*
	103.2	56.8	82.8	69.7	77.0	62.0
→3)-α-l-6dTal*p*-(1→ **B**	*4.97*	*3.74*	*3.87*	*3.88*	*4.28*	*1.23*
	103.2	71.1	72.0	70.3	68.8	16.7
→2)-α-l-Rha*p*-(1→ **C**	*5.08*	*4.01*	*3.95*	*3.48*	*3.75*	*1.30*
	97.7	79.8	71.1	73.5	70.5	18.0
→2,3)-α-l-Rha*p*-(1→ **D**	*5.16*	*4.40*	*3.92*	*3.56*	*3.75*	*1.25*
	102.4	79.3	81.0	72.0	70.5	17.8^*f*^
→4)-β-d-Glc*p*A-(1→ **E**	*4.78*	*3.39*	*3.77*	*3.81*	*3.95*	
	104.8	74.5	77.2	78.3	76.1	174.1
→2)-α-d-Glc*p*-(1→ **F**	*5.42*	*3.55*	*3.72*	*3.68*	*3.54*	*3.69, 3.77*
	100.0	78.2	72.8	72.4	73.0	61.1
α-l-Rha*p*-(1→ **G**	*4.86*	*3.98*	*3.76*	*3.44*	*4.00*	*1.25*
	102.0	71.7	71.5	73.3	70.3	17.9^*f*^
Oligosaccharide **5** (at 40°C)^*c*^						
→3)-α-d-Glc*p*NAc **Aα**	*5.15*	*4.03*	*3.82*	*3.55*	*3.89*	*3.80, 3.86*
	92.4	54.9	80.7	69.8	73.1	62.0
→3)-β-d-Glc*p*NAc-(1→ **Aβ**	*4.75*	*3.79*	*3.63*	*3.52*	*3.49*	*3.79, 3.92*
	95.7	57.8	83.1	69.8	77.3	62.1
→3)-α-l-6dTal*p*-(1→ **B**	*4.98*^*d*^	*3.73*	*3.88*	*3.92*	*4.30*	*1.24*
	103.2^*e*^	71.3	72.1	70.3	68.8	16.8
→2)-α-l-Rha*p*-(1→ **C**	*5.11*	*4.04*	*3.98*	*3.49*	*3.78*	*1.32*
	97.8	80.3	71.1	73.4^*g*^	70.6^*h*^	18.1
→2)-α-l-Rha*p*-(1→ **D**	*5.27*	*4.16*	*3.90*	*3.50*	*3.78*	*1.27*
	102.4	81.4	71.2	73.6^*g*^	70.3^*h*^	17.8^*i*^
→4)-β-d-Glc*p*A-(1→ **E**	*4.62*	*3.44*	*3.77*	*3.80*	*3.78*	
	105.5	74.7	77.4	78.0	78.3	n.f.
→2)-α-d-Glc*p*-(1→ **F**	*5.43*	*3.55*	*3.74*	*3.79*	*3.54*	*3.70, 3.81*
	99.7	78.2	72.9	72.2	73.2	61.2
α-l-Rha*p*-(1→ **G**	*4.87*	*4.00*	*3.79*	*3.46*	*4.03*	*1.27*
	102.1	71.8	71.5	73.3	70.3	18.0^*i*^

a ^1^H NMR chemical shifts are italicized. n.f., not found.

b Chemical shifts for the *N*-acetyl groups are δ_H_ 2.02, δ_C_ 23.5 (Me), and 175.5 (CO).

c Chemical shifts for the N-acetyl groups are δ_H_ 2.06, δ_C_ 23.3 (**Aα**), 23.5 (**Aβ**) (both Me), and 176.3 (CO).

d When linked to **Aα**; δ 4.96 when linked to **Aβ**.

e When linked to **Aα**; δ 103.3 when linked to **Aβ**.

f Assignment could be interchanged.

g Assignment could be interchanged.

h Assignment could be interchanged.

i Assignment could be interchanged.

**TABLE 8:** ^1^H and ^13^C NMR chemical shifts of the K32 CPS of A. baumannii LUH5549 and oligosaccharide **6** derived by depolymerization of the CPS with phage depolymerase APK32_gp46^*a*^

Residue	^1^H and ^13^C chemical shifts (**δ**, ppm)					
	*H-1* C-1	*H-2* C-2	*H-3* C-3	*H-4* C-4	*H-5* C-5	*H-6a, 6b* C-6
CPS (at 65°C) (19)						
→3)-β-d-Gal*p*NAc-(1→ **A**	*4.54* 104.1	*3.83* 52.5	*3.89* 81.3	*4.06* 69.2	*3.59* 75.9	*3.74, 3.82* 62.6
→4)-β-d-Glc*p*A-(1→ **B**	*4.58* 105.3	*3.40* 73.5	*3.64* 75.2	*3.84* 81.2	*4.01* 74.8	172.1
→3,4)-β-d-Gal*p*NAc-(1→ **C**	*4.53* 102.9	*4.06* 52.8	*3.84* 79.0	*4.22* 74.9	*3.73* 77.0	*3.64, 3.68* 61.6
→6)-α-d-Glc*p*NAc-(1→ **D**	*4.86* 98.3	*3.90* 55.3	*3.87* 71.9	*3.71* 70.9	*4.34* 72.0	*4.06, 4.32* 69.3
β-d-Glc*p*-(1→ **E**	*4.47* 104.0	*3.35* 74.5	*3.49* 77.2	*3.40* 71.2	*3.43* 77.2	*3.72, 3.91* 62.4
Oligosaccharide **6** (at 30°C)						
→3)-α-d-Gal*p*NAc **Aα**	*5.22* 92.4	*4.29* 50.2	*4.01* 78.6	*4.20* 69.8	*4.13* 71.5	*3.74, 3.74* 62.5
→3)-β-d-Gal*p*NAc **Aβ**	*4.69* 96.4	*3.99* 53.6	*3.82* 81.5	*4.13* 69.1	*3.69* 76.2	*3.74, 3.76* 62.3
→4)-β-d-Glc*p*A-(1→ **B(α)**^*b*^	*4.57* 105.3	*3.38* 73.9	*3.59* 75.1	*3.78* 81.2	*3.69* 77.8	175.5
→4)-β-d-Glc*p*A-(1→ **B(β)**^*c*^	*4.51* 105.5	*3.38* 73.8	*3.59* 75.0	*3.78* 81.2	*3.69* 77.8	175.5
→4)-β-d-Gal*p*NAc-(1→ **C**	*4.55* 102.6	*3.97* 53.3	*3.79* 71.7	*3.98* 77.5	*3.74* 76.7	*3.74, 3.74* 61.5
→6)-α-d-Glc*p*NAc-(1→ **D**	*4.90* 99.5	*3.95* 55.2	*3.87* 71.7	*3.64* 70.8	*4.31* 72.3	*3.91, 4.07* 69.2
β-d-Glc*p*-(1→ **E**	*4.48* 103.9	*3.32* 74.4	*3.50* 76.9	*3.40* 70.9	*3.44* 77.1	*3.73, 3.91* 62.0

a ^1^H NMR chemical shifts are italicized. Chemical shifts for the *N*-acetyl groups are δ_H_ 1.96–2.09, δ_C_ 23.1–23.8 (Me), and 175.1–176.3 (CO).

b Linked to **Aα**.

c Linked to **Aβ**.

**TABLE 9:** ^1^H and ^13^C NMR chemical shifts of the K89 CPS of A. baumannii LUH5552 and oligosaccharide **8** derived by depolymerization of the CPS with depolymerase APK89_gp46^*a*^

Monosaccharide residue	^1^H and ^13^C chemical shifts (**δ**, ppm)					
	*H-1* C-1	*H-2* C-2	*H-3* C-3	*H-4* C-4	*H-5* C-5	*H-6a, 6b* C-6
CPS (at 55°C)						
→3)-β-d-Glc*p*NAc-(1→ **A**	*4.59* 103.9	*3.69* 55.3	*3.76* 79.5	*3.59* 72.8	*3.42* 77.1	*3.75, 3.93* 62.2
→3,4)-α-d-Gal*p*NAc-(1→ **B**	*5.42* 98.5	*4.39* 50.1	*3.82* 77.5	*4.36* 76.0	*3.90* 73.0	*3.84, 3.86* 61.2
→6)-α-d-Glc*p*-(1→ **C**	*5.02* 99.9	*3.51* 73.1	*3.84* 73.4	*3.82* 70.0	*4.20* 71.8	*4.03, 4.15* 69.0
→2)-β-d-Fuc*p*3NAc-(1→ **D**	*4.61* 103.7	*3.84* 75.7	*4.15* 55.8	*3.68* 71.8	*3.85* 73.0	*1.28* 17.0
β-d-Glc*p*-(1→ **E**	*4.58* *103.5*	*3.32* 74.8	*3.48* 77.1	*3.38* 71.2	*3.43* 77.5	*3.74, 3.94* 62.5
Oligosaccharide **8** (at 35°C)						
→3)-α-d-Glc*p*NAc **Aα**	*5.17* 92.3	*4.01* 53.8	*3.92* 77.6	*3.70* 72.2	*3.88* 72.6	*3.74, 3.90* 61.6
→3)-β-d-Glc*p*NAc **Aβ**	*4.76* 95.9	*3.75* 56.6	*3.74* 79.8	*3.67* 72.2	*3.47* 77.1	*3.79, 3.84* 61.8
→4)-α-d-Gal*p*NAc-(1→ **B**	*5.44*^*b*^ 98.9^*b*^	*4.25* 51.2^*c*^	*3.92* 72.8	*4.11* 79.3	*3.93* 72.5	*3.87* 61.2
→6)-α-d-Glc*p*-(1→ **C**	*4.98* 101.6	*3.55* 73.2	*3.82* 73.8	*3.76* 70.1	*4.17* 72.3	*3.92, 4.04* 69.1
→2)-β-d-Fuc*p*3NAc-(1→ **D**	*4.58* 103.6	*3.81* 75.6	*4.15* 55.8	*3.66* 71.8	*3.87* 73.1	*1.24* 16.6
β-d-Glc*p*-(1→ **E**	*4.57* 103.6	*3.30* 74.6	*3.47* 76.9	*3.37* 71.1	*3.41* 77.4	*3.72, 3.93* 62.4

a ^1^H NMR chemical shifts are italicized. Chemical shifts for the *N*-acetyl groups are δ_H_ 1.99–2.08, δ_C_ 23.2–23.9 (Me), and 174.6–175.7 (CO).

b Linked to **Aα**.

c Linked to **Aβ**.

**TABLE 10:** ^1^H and ^13^C NMR chemical shifts of the K93 CPS from A. baumannii B11911 and oligosaccharide **10** derived by depolymerization of the CPS with depolymerase APK93_gp43^*a*^

Monosaccharide residue	^1^H and ^13^C chemical shifts (**δ**, ppm)								
	*H-1*, C-1	*H-*2, C-2	*H-3 (3ax, 3eq)*, C-3	*H-4*, C-4	*H-5*, C-5	*H-6 (6a, 6b)*, C-6	*H-7*, C-7	*H-8*, C-8	*H-9*, C-9
CPS (at 65°C) (23)									
→3)-β-d-Gal*p*NAc-(1→ **A**	*4.74*	*4.04*	*3.88*	*4.11*	*3.66*	*3.75, 3.75*			
	103.9	53.0	81.1	69.7	76.0	62.5			
→3,6)-β-d-Gal*p*-(1→ **B**	*4.48*	*3.63*	*3.72*	*4.17*	*3.87*	*3.69, 3.69*			
	106.0	71.4	82.7	69.9	73.7	67.9			
→6)-α-d-Gal*p*-(1→ **C**	*4.97*	*3.83*	*3.80*	*3.95*	*4.05*	*3.56, 3.95*			
	100.1	69.7	70.7	70.7	70.8	65.1			
β-Pse*p*5Ac7*R*Hb-(2→ **D**			*1.57, 2.50*	*3.87*	*4.18*	*3.96*	*4.00*	*4.07*	*1.22*
	174.1	102.6	37.1	68.1	49.9	74.7	54.9	70.9	19.2
Oligosaccharide **10** (at 60°C)									
→3)-α-d-Gal*p*NAc **Aα**	*5.23*	*4.31*	*4.02*	*420*	*4.11*	*3.74, 3.79*			
	92.6	50.4	78.6	70.2	71.6	62.7			
→3)-β-d-Gal*p*NAc **Aβ**	4.71	*3.98*	*3.85*	*4.13*	*3.67*	*3.74, 3.74*			
	96.5	54.0	81.5	69.6	76.2	62.5			
→6)-β-d-Gal*p*-(1→ **B(α)**^*b*^	*4.52*	*3.56*	*3.66*	*3.98*	*3.87*	*3.70, 3.70*			
	106.1	72.0	74.0	69.9	73.9	68.0			
→6)-β-d-Gal*p*-(1→ **B(β)**^*c*^	*4.47*	*3.56*	*3.66*	*3.98*	*3.87*	*3.70, 3.70*			
	105.9	72.1	74.0	70.0	74.1	67.9			
→6)-α-d-Gal*p*-(1→ **C**	*4.98*	*3.84*	*3.82*	*3.97*	*3.97*	*3.57, 3.95*			
	100.0	69.6	70.8	70.8	70.6	65.1			
β-Pse*p*5Ac7RHb-(2→ **D**			*1.59, 2.51*	*3.87*	*4.18*	*3.98*	*4.00*	*4.07*	*1.22*
	174.6	n.f.	37.1	68.0	49.9	74.7	54.9	70.8	19.0

a Hb, (*R*)-3-hydroxybutanoyl.^1^H NMR chemical shifts are italicized. Chemical shifts for the *N*-acetyl groups are δ_H_ 2.02–2.04, δ_C_ 23.5–23.8 (Me); for NHb, δ_C_ 46.4 (C-2), 66.3 (C-3), 23.6 (C-4), δ_H_ 2.35 (H-2), 4.16 (H-3), 1.22 (H-4); for CO groups, δ_C_ 175.7–176.2. n.f., not found.

b Linked to **Aα**

c Linked to **Aβ**.

**TABLE 11:** ^1^H and ^13^C NMR chemical shifts of the K116 CPS from A. baumannii MAR303 and oligosaccharide **14** derived by depolymerization of the CPS with phage depolymerase APK116_gp43^*a*^

Monosaccharide residue	^1^H and ^13^C chemical shifts (**δ**, ppm)					
	*H-1*, C-1	*H-2*, C-2	*H-3*, C-3	*H-4*, C-4	*H-5*, C-5	*H-6a, 6b*, C-6
CPS^*b*^ (at 55°C) (24)				
→3)-β-d-Gal*p*NAc-(1→ **A**	*4.66* 104.5	*4.11* 52.9	*3.85* 81.6	*4.16* 69.4	*3.70* 76.0	*3.78,3.78* 62.7
→6)-β-d-Gal*p*-(1→ **B**	*4.48* 106.0	*3.56* 72.0	*3.63* 73.8	*4.02* 69.7	*3.70* 76.0	*3.83, 3.83* 67.7
→3,4)-α-d-Gal*p*-(1→ **C**	*4.96* 100.4	*3.74* 68.7	*3.91* 80.9	*4.37* 77.6	*3.94* 71.9	*3.74, 3.81* 62.4
→6)-α-d-Gal*p*NAc-(1→ **D**	*4.92* 103.1	*3.93* 53.6	*3.78* 72.1	*3.97* 69.2	*3.85* 74.8	*3.92, 4.08* 70.4
β-d-Glc*p*-(1→ **E**	*4.51* 104.1	*3.30* 74.4	*3.50* 77.0	*3.40* 71.1	*3.49* 77.2	62.1 *3.75, 3.93*
Oligosaccharide^*c*^ **14** (at 45°C)				
→3)-α-d-Gal*p*NAc **A^α^**	*5.23* 92.4	*4.31* n.f.	*4.02* 78.3	*4.20* 69.9	*4.12* 71.4	*3.78* 62.7
→3)-β-d-Gal*p*NAc **A^β^**	*4.72* 96.2	*3.97* 53.9	*3.87* 81.5	*4.14* 69.3	*4.14* 78.3	*3.87, 3.87* 62.7
→6)-β-d-Gal*p*-(1→ **B**	*4.46* 105.8	*3.54* 71.8	*3.64* 73.8	*3.96* 69.9	*3.70* 75.9	*3.87, 3.87* 68.0
→4)-α-d-Gal*p*-(1→ **C**	*4.96* 99.8	*3.72* 69.7	*3.92* 70.8	*4.35* 77.6	*3.91* 71.7	*3.76, 3.81* 62.3
→6)-α-d-Gal*p*NAc-(1→ **D**	*4.90* 103.0	*3.91* 53.6	*3.76* 72.1	*3.96* 69.9	*3.82* 75.2	*3.96, 4.07* 70.4
β-d-Glc*p*-(1→ **E**	*4.50* 104.1	*3.30* 74.4	*3.50* 76.9	*3.40* 70.9	*3.47* 77.2	*3.75, 3.93* 62.1

a ^1^H NMR chemical shifts are italicized.

b Chemical shifts for the *N*-acetyl groups are δ_H_ 2.03–2.07, δ_C_ 23.7–24.1 (CH_3_), and 175.7–176.3 (CO).

c Chemical shifts for the *N*-acetyl groups are δ_H_ 2.03–2.06, δ_C_ 23.6, 23.7 (CH_3_), and 176.0, 176.2 (CO).

The ^13^C NMR chemical shifts of all but two monosaccharide residues in the K unit monomers were essentially the same in the oligosaccharides and the corresponding CPSs, whereas those of the residues at the reducing and nonreducing ends of the oligosaccharides were different. On this basis, the glycosidic linkages that were cleaved in the CPSs by the TSDs could be identified (Table 3).

As expected, the monosaccharides that occupy the reducing end of the oligosaccharides (d-GlсNAc in **5** and **8**, d-GalNAc in **2**, **6**, **10**, and **12**) were present in two anomeric forms (**α** and **β**), which showed in the ^13^C NMR spectrum the C-1 signals characteristic for nonlinked monosaccharides at δ 92.3–92.6 and 95.7–96.5, respectively (Tables 5 to 11). In oligosaccharide **1**, the reducing end was occupied by a legionaminic acid derivative (Leg*p*5Ac7R), as followed from a displacement of the C-6 signal of this monosaccharide from δ 73.1 in the CPS to δ 70.9 in **1** (Table 5). This difference reflected conversion, upon cleavage of the glyosidic linkage of the α-linked Leg*p*5Ac7R residue with the axial carboxyl group, into a more stable β-anomer with the equatorial carboxyl group (26).

Furthermore, the ^13^С NMR chemical shifts of unit **C** (β-Gal) in **1** were typical of the corresponding nonsubstituted residues (Table 5) (27). Hence, this monosaccharide, which was substituted in the corresponding initial CPS, was located at the nonreducing end in **1** (Fig. 5). Unit **B** (β-Gal in **10,** α-GalNAc in **8**), unit **C** (β-GalNAc in **6**), and unit **D** (α-Rha in **5**, β-GalNAc in **2**) were disubstituted in the CPSs but became monosubstituted in the corresponding oligosaccharides (Fig. 5–7). This conclusion followed from significant upfield displacements (by 7.3 to 10.2 ppm) of the signals for C-4 of unit **D** in **2** and C-3 of units **B**, **C,** or **D** in **5**, **6**, **8,** and **10**, compared with their positions in the corresponding CPSs (Tables 5 to 10). Therefore, these carbons that were linked in the CPSs became nonlinked in the oligosaccharides. These data defined the structures of the oligosaccharides obtained by depolymerization of the CPSs (Fig. 5–7) and, as a result, identified the linkages that were cleaved by phage depolymerases (Table 3).

The CPSs of strains ACICU and B11911, having the same main chains consisting of disaccharide repeats but different side chains (Fig. 7), were cleaved with the same depolymerase APK2_gp43, in the same manner, by the β-d-Gal*p*NAc-(1→3)-β-d-Gal*p* (**A→B**) linkage (Fig. 7). The same linkage was also cleaved by a TSD of bacteriophage φAB6, in the CPS of A. baumannii 54149 (6), having the same structure as the CPS of strain ACICU.

The ^1^H and ^13^C NMR spectra of the larger oligosaccharides **3**, **7**, **9**, and **11** showed two series of signals, one being the same as in the corresponding K unit monomers **2**, **6**, **8**, and **10** and the other being the same as in the corresponding CPS K units. Based on these and HR ESI-MS data (Table 4), it was concluded that these oligosaccharides represented K unit dimers having the structures shown in Fig. 5 to 7. A comparison of the NMR spectra of oligosaccharide **4** and those of the CPS of strain NIPH615 (22), combined with the HR ESI-MS data (Table 4), showed that **4** was a K unit dimer with a β-d-Glc*p*NAc residue at the reducing end (Fig. 5).

## DISCUSSION

In the scope of phage application for the development of preparations to control multidrug-resistant A. baumannii strains, a comprehensive characterization of prospective candidates to be included in these preparations is required. One of the important characteristics for the applied usage of virulent phages is a host range—the ability of a phage to demonstrate a lytic activity against diverse, clinically important bacterial strains that circulate in the area of estimated application. Taking into account that the CPSs are the primary receptors for depolymerase-carrying A. baumannii phages (5–9), we believe that the range of their lytic spectra depends on the prevalence of A. baumannii strains with appropriate CPS structures.

High diversity and variability of the CPS structures imply the existence of the same variety of phage depolymerases that can specifically recognize and cleave different CPSs. Therefore, preparations (“cocktails”) comprising different phages with the corresponding CPS-recognizing proteins are required to eliminate bacteria of different capsular types within the same species. Certainly, in rare cases, several variants of K loci can be responsible for the synthesis of CPSs of the same structure (for example, KL3/22), or some phages like APK2, described in this work, are able to infect bacteria belonging to different K types. However, a genetic exchange within the population of A. baumannii strains and a rapid appearance of new capsular types are ongoing all the time. Therefore, the constant search for new phages with different K specificity is necessary.

Nowadays, phages that are specific to K1 (phage P1) (8), K2 (phages φAB6 and vB_AbaP_B3) (6, 8), K9 (phages vB_AbaP_B1, AM24, and BS46) (8, 9, 28), K19 (phages Fri1 and AS11) (7), K27 (phage AS12) (7), K45 (phage vB_AbaM_B9, which also performed lysis from without in a K30 strain) (29), and K91 (phage AP22) (13) capsular types of A. baumannii have been described. For some other depolymerase-carrying phages, there is no information as to which capsular type their A. baumannii host strains belong (30–32).

In this work, eight novel A. baumannii bacterial viruses and depolymerases, encoded in their genomes, were characterized. All phages share a similar genome organization with previously reported viruses of the *Friunavirus* genus, with the most variable region falling into gene regions encoding CPS-recognizing/degrading domains of tailspikes or structural depolymerases. Seven of the phages, namely, APK32, APK37, APK44, APKK48, APKK87, APKK89, and APK116, are the first reported viruses specific to A. baumannii strains of the K32, K37, K44, K48, K87, K89, and K116 capsular types, respectively. Phage APK2 was shown to infect A. baumannii K2 like the earlier described phages φAB6 (6) and vB_AbaP_B3 (8) and also A. baumannii K93 like phage APK93. Interestingly, phages APK2 and APK93 were initially isolated from different samples in 2018 and were given their names because of their isolation on the K2 and K93 bacterial lawns, respectively. Genome sequencing of these phages revealed that they are close homologs and that their receptor-recognizing/binding proteins or TSDs are completely identical. Therefore, these phages can be recognized as closely related variants.

Analysis of oligosaccharide products obtained by degradation of the A. baumannii CPSs by recombinant depolymerases APK2_gp43, APK32_gp46, APK37_gp44, APK44_gp44, APK48_gp43, APK87_gp48, APK89_gp46, and APK116_gp43 showed that all TSDs studied were specific glycosidases that cleaved the CPSs by the hydrolytic mechanism, to give a monomer or/and an oligomer(s) of the K units. The specific interaction of the APK2_gp43 depolymerase with both K2 and K93 CPSs was suggested to be due to the similarity of their K units, which have identical main chains and differ only in their side chain structures (Fig. 7). Particularly identical are the linkages between the K2 and K93 K units that are cleaved by the APK2_gp43 depolymerase.

Worthy of note is that phages APK2, φAB6, and vB_AbaP_B3, with the same specificity to K2 A. baumannii strains, were independently isolated in remote geographic locations: Russia, China, and Portugal, respectively. The same situation has been observed for phages that infect A. baumannii strains of the K9 capsular type. These findings suggest that representatives of these K types are widely spread around the world and that the phages should adopt their receptor-binding/recognizing proteins to successfully infect them.

In conclusion, the comprehensive characterization of new lytic phages and phage-encoded TSDs expand our knowledge of virus-bacterial host interaction strategies. Numerous A. baumannii phages isolated by different research groups, in various geographic locations, have demonstrated “broad” or “narrow” lytic spectra. As each TSD specifically recognizes and enzymatically digests only certain CPS, we declare that the spectrum range of a depolymerase-carrying A. baumannii phage depends on the distribution of A. baumannii strains with appropriate CPS structure among all those tested in each particular case. That means the different phages, independently on broad or narrow spectra, could be efficiently used as antibacterial tools, depending on the clinical situation.

## MATERIALS AND METHODS

### Phage isolation, propagation, and purification.

For phage isolation, a collection of various A. baumannii strains with defined CPS structures (listed in Table 1), kindly provided by the members of research groups from different countries (see Acknowledgements), was used. Bacteriophages were isolated from sewage and environmental (river water) samples, in accordance with a previously reported procedure (33). The samples were cleared by low-speed centrifugation (7,000 × *g* for 30 min), the supernatants supplemented with LB medium were incubated for 16 to 18 h in the presence of growing A. baumannii strains belonging to capsular types of interest at 37°C, and then chloroform was added. Bacterial debris was pelleted by centrifugation at 7,000 × *g* for 30 min, followed by filtration of the supernatants through 1.20- and 0.45-µm-pore-size membrane filters. The purified filtrates were concentrated by ultracentrifugation at 85,000 × *g* at 4ºС for 2 h. The spot test method, as well as the plaque assay (34), was used to screen for the presence of lytic phage activity in the resulting concentrated preparations. The plates were incubated overnight at 37°C and examined for zones of lysis or plaque formation.

Single plaques formed on the lawns of sensitive A. baumannii strains were picked up in SM buffer (10 mM Tris-HCl, pH 7.5, 10 mM MgSO_4_, 100 mM NaCl) and replated three times, in order to obtain pure phage stock.

A. baumannii strain ACICU belonging to the K2 capsular type was used as the host for phage APK2 propagation, and A. baumannii strains NIPH70 (K44), NIPH146 (K37), NIPH615 (K48), LUH5547 (K87), LUH5549 (K32), LUH5552 (K89), and MAR303 (K116) were used as the hosts for phages APK44, APK37, APK48, APK87, APK32, APK89, and APK16 propagation, respectively.

The phages were propagated using a liquid culture of the A. baumannii host strains (optical density at 600 nm [OD_600_] of 0.3) at a multiplicity of infection (MOI) of 0.1.

The phage particles were precipitated by polyethylene glycol (PEG) 8000 (to a final concentration of 10% and 500 mM NaCl). The final purification was executed by cesium chloride density gradient centrifugation at 100,000 × *g* (SW50.1 Ti rotor; Beckman Coulter Inc., Brea, CA, USA) for 24 h (35).

### Phage host specificity determination.

The host specificity of the phages was tested against A. baumannii strains belonging to 56 different K types (presented in Table 1), using the double-layer method (34). For this, 300 µl of A. baumannii bacterial cultures grown in LB medium at 37°C to an OD_600_ of 0.3 was mixed with 4 ml of soft agar (LB broth supplemented with 0.6% agarose). The mixture was plated onto the nutrient agar. Then, the phage suspensions (∼10^9^ PFU per ml) or purified recombinant depolymerases, and their several dilutions, were spotted on the soft agar lawns and incubated at 37°C for 18 to 24 h.

### Phage infection inhibition assay.

The effect of purified TSD proteins APK2_gp43, APK32_gp46, APK37_gp44, APK44_gp44, APK48_gp43, APK87_gp48, APK89_gp46, and APK116_gp43 on the phage infection of A. baumannii ACICU, LUH5549, NIPH146, NIPH70, NIPH615, LUH5547, LUH5552, and MAR303 host cells was assessed as following. A titer of 2 × 10^6^ PFU/ml for phages APK2, APK32, APK37, APK44, APK48, APK87, APK89, and APK116 was chosen for the competition experiments. A. baumannii host strains were grown in LB medium at 37°C to an OD_600_ of 0.3. Then, 20 μM concentrations of corresponding TSD solutions were added to 100-μl aliquots of the cell cultures and incubated for 20 min at 37°C. One-hundred-microliter aliquots of the A. baumannii host cells without anything and with 20 μM BSA incubated for 20 min at 37°C served as controls. After the incubation, several dilutions of corresponding phages and 4 ml of soft agar were added to the mixtures and plated onto the nutrient agar. The plates were incubated overnight at 37°C and assayed for the number of lysis plaques. The experiment was performed in triplicate.

### Electron microscopy.

The phage was examined by negative-contrast electron microscopy, using the following procedure. Three microliters of purified and concentrated phage preparation was applied to the carbon-coated 400 mesh copper grids and subjected to glow discharge using the Emitech K100X apparatus (Quorum Technologies, UK). Grids were then negatively stained with 1% uranyl acetate for 30 s, air dried, and analyzed using a JEOL JEM-2100 200-kV transmission electron microscope. Images of negatively stained phage particles were taken with a Gatan Ultrascan 1000XP charge-coupled-device (CCD) camera (14  µm pixels) and Gatan Digital Micrograph software with the following parameters: ×30,000 magnification, 0.5 to 1 µm defocus, 40-µm objective aperture, 2k by 2k pixel size unbinned image size, 3.4-Å pixel size. 

### DNA isolation and sequencing.

Phage genomic DNAs were isolated from concentrated and purified high-titer phage stocks by incubation in 0.5% SDS, 20 mM EDTA, and 50 µg/ml proteinase K at 56°C for 1 to 3 h. The DNAs were extracted with phenol-chloroform and then precipitated with ethanol supplemented with sodium acetate (35).

Genome sequencing was performed on the MiSeq platform using a Nextera DNA library preparation kit (Illumina, San Diego, CA). The generated reads were assembled *de novo* into a single contig using SPAdes v.3.13 (36). Further, the resulting sequences were checked by mapping reads against the assemblies with DNASTAR's Lasergene sequence analysis software, version 11.1.0 (DNASTAR, Inc., Madison, WI, USA) (37). Direct terminal repeats were determined as regions of greater coverage of sequencing reads mapped on assembled viral genome contigs. The ends were then verified directly by Sanger sequencing with outward-directed primers located inside and outside putative repeats.

### Phage genome analysis.

Potential open reading frames (ORFs) were identified with the RAST automated annotation engine (38). Predicted proteins were searched against the NR (nonredundant) database of the NCBI and HHpred profile-profile search (39). The presence of tRNAs in the genome sequence was determined using tRNAscan-SE, version 2.0 (40). Comparative analysis of DNA genome sequences was performed using Easyfig (41).

### Cloning, expression, and purification of the recombinant depolymerases.

The DNA fragments corresponding to the deletion mutants lacking N‐terminal domains of putative podophage depolymerases were amplified by PCR, using oligonucleotide primers indicated in Table 12, and cloned into the pTSL plasmid (GenBank accession no. KU314761) (42).

**TABLE 12:** Oligonucleotide primers used in this study for cloning of phage depolymerases

Primer	Sequence (5′–3′)^*a*^	Restriction site for:
APK2_dep_F	ataGGATCCgaagaggctgctcagcaaac	BamHI
APK2_dep_R	ataAAGCTTaaaatccagataccacagtaa	HindIII
APK32_dep_F	ataGGATCCcaagcacaagaggctagcg	BamHI
APK32_dep_R	ataCTCGAGttaatctggattaaaaataatatcta	XhoI
APK37_dep_F	ataGGATCCgaagcaagtcaagctgctca	BamHI
APK37_dep_R	ataAAGCTTaggataatacatggctaatgt	HindIII
APK44_dep_F	ataGGATCCgaagctagtgaagctgctca	BamHI
APK44_dep_R	ataAAGCTTatttgaaacgatacttaaatcgt	HindIII
APK48_dep_F	ataGGATCCgaggaagctgcacaggttac	BamHI
APK48_dep_R	ataCTCGAGttaatttactactacactacctag	XhoI
APK87_dep_F	ataGGATCCgaagatgcttctgatgcactt	BamHI
APK87_dep_R	ataAAGCTTaaataagtttaataagccctcg	HindIII
APK89_dep_F	ataGGATCCgaagctagtgaagctgctca	BamHI
APK89_dep_R	ataAAGCTTatttaatgtatcgagcgtcac	HindIII
APK116_dep_F	ataGGATCCaacgcagcagaagtagctg	BamHI
APK116_dep_R	ataAAGCTTagtctttaagatttatgtcgt	HindIII

a Uppercase letters indicate restriction endonuclease recognition sites.

Expression vectors were transformed into chemically competent Escherichia coli B834(DE3) cells. Protein expression was performed in LB medium supplemented with ampicillin at 100 µg/ml. Transformed cells were grown at 37°C until the optical density reached a value of 0.6 at 600 nm. The medium was cooled to the temperature of 18°C, followed by expression induction by the addition of isopropyl-1-thio-β-d-galactopyranoside (IPTG) to a final concentration of 0.5 to 1.0 mM. After further incubation at 18°C overnight (approximately 16 h), the cells were harvested by centrifugation at 10,000 × *g* for 15 min, at 4°C. The cell pellets were resuspended in buffer A (20 mM Tris [pH 8.0], 300 mM NaCl, 1/50 of the original cell volume), frozen, thawed, and disintegrated by sonication (Branson Ultrasonic, Danbury, CT, USA). The cell debris was removed by centrifugation at 15,000 × *g* at 4°C for 20 min. The supernatant was applied to 5-ml Ni^2+^-charged GE HisTrap columns (GE Healthcare Life Sciences, Chicago, IL, USA) equilibrated with buffer A, and the proteins were eluted with a 50-to-200 mM imidazole linear gradient in buffer A. Fractions containing the target protein were pooled and digested with tobacco etch virus (TEV) protease for 16 h at 20°C at a protease/protein ratio of 1/100 (wt/wt) to remove the His tag. The reaction mixture was simultaneously dialyzed against 10 mM Tris-HCl (pH 8.0) containing 1.0 mM 2-mercaptoethanol.

The cleaved proteins were clarified by filtration, loaded onto an ion-exchange MonoQ 10/100 GL column (GE Healthcare), and eluted with a 0-to-650 mM NaCl gradient in 20 mM Tris-HCl (pH 8.0) buffer. Protein-containing fractions were combined and concentrated to ∼5 ml using Sartorius ultrafiltration devices (Sartorius AG, Germany), with a molecular-weight cutoff of 50,000 Da.

### Isolation and purification of the capsular polysaccharides.

A. baumannii ACICU, NIPH70, NIPH146, NIPH615, LUH5547, lUH5549, LUH5552, B11911, and MAR303 were cultivated in 2TY medium (16 g Bacto tryptone, 10 g Bacto yeast extract, and 5 g NaCl, adjusted to 1 liter with distilled H_2_O) for 16 h. Bacterial cells were harvested by centrifugation (10,000 × *g*, 20 min), washed with phosphate-buffered saline, suspended in aqueous 70 % acetone, precipitated, and dried in air.

Capsular polysaccharides (CPSs) were isolated by extraction of A. baumannii cells with 45% aqueous phenol for 30 min at 65 to 68°C (43). The extract was cooled and dialyzed without layer separation; then, insoluble contaminations were removed by centrifugation (12,000 × *g*, 20 min), and CPS preparations were purified as described previously (44). Briefly, aqueous 50% CCl_3_CO_2_H was added to a CPS solution in water at 4°C, a precipitate was removed by centrifugation, and the supernatant was dialyzed with distilled water and freeze-dried. The crude CPS preparations were heated with 2% HOAc (100°C, 3 h), and a lipid precipitate was removed by centrifugation (12,000 × *g*, 20 min). Purified CPS samples were isolated from the supernatant by gel permeation chromatography on a XK 26-mm (depth) by 70-cm (height) column (gel layer, 560 mm) (GE Healthcare) of Sephadex G-50 Superfine (Amersham Biosciences, Sweden) in 0.05 M pyridinium acetate buffer, pH 4.5. The flow rate was 0.5 ml min^−1^; elution was monitored with a differential refractometer (Knauer, Germany). Control of retention of the intact structure upon mild acid treatment was performed by NMR spectroscopy.

### Cleavage of the CPSs with tailspike depolymerases.

Purified CPSs were solubilized in 20 mM Tris-HCl (pH 7.5) buffer, and 200 to 500 µg of the corresponding TSD proteins was added for digestion. The reaction mixture was stored at 37°C for 16 h.

CPS digestion products were fractionated by gel permeation chromatography on a XK 16-mm (depth) by 100-cm (height) column (gel layer, 800 mm) (GE Healthcare) of Fractogel TSK HW-40S (Toyo Soda, Japan) in 1% acetic acid at a flow rate of 0.5 ml min^−1^ and monitored as described above.

### NMR spectroscopy.

Samples were deuterium exchanged by freeze-drying from 99.9% D_2_O and then examined as solution in 99.95% D_2_O. NMR spectra were recorded on a Bruker Avance II 600 MHz spectrometer (Bruker, Germany) at 30 to 60°C, as indicated in Tables 5 to 11. Sodium 3-trimethylsilylpropanoate-2,2,3,3-d_4_ (δ_H_ 0, δ_C_ −1.6) was used as an internal reference for calibration. Two-dimensional ^1^H-^1^H correlation spectroscopy (COSY), ^1^H^1^H total correlation spectroscopy (TOCSY), ^1^H-^1^H rotating-frame Overhauser effect spectroscopy (ROESY), ^1^H-^13^C heteronuclear single-quantum coherence (HSQC), and ^1^H-^13^C heteronuclear multiple-bond correlation (HMBC) experiments were performed using standard Bruker software and were used for assignment of ^1^H and ^13^C NMR chemical shifts (25). The Bruker TopSpin 2.1 program was used to acquire and process the NMR data. A MLEV-17 spin-lock time of 60 ms and a mixing time of 200 ms were used in TOCSY and ROESY experiments, respectively. A 60-ms delay was used for evolution of long-range couplings to optimize the ^1^H,^13^C HMBC experiments for coupling constant *J*_H,C_ 8 Hz.

### Mass spectrometry.

High-resolution electrospray ionization mass spectrometry (HR ESI-MS) (45) was performed in the negative ion mode using a micrOTOF II instrument (Bruker Daltonics). Oligosaccharide samples (∼50 ng µl^−1^) were dissolved in a 1:1 (vol/vol) water-acetonitrile mixture and injected with a syringe at a flow rate of 3 µl min^−1^. The capillary entrance voltage was set at 3,200 V, and the interface temperature was set at 180°C. Nitrogen was used as the drying gas. The mass range was from *m/z* 50 to 3,500. Internal calibration was carried out with ESI calibrant solution (Agilent).

### Data availability.

The genome sequences of vB_AbaP_APK2, APK32, APK37, APK44, APK48, APK87, APK89, and APK116 phages were deposited in GenBank under accession numbers MK257719, MK257722, MK257723, MN604238, MN294712, MN604239, MN651570, and MN807295, respectively (Table 2).
